# Near-Full Genome Characterisation of Two Natural Intergenotypic 2k/1b Recombinant Hepatitis C Virus Isolates

**DOI:** 10.1155/2011/710438

**Published:** 2011-05-15

**Authors:** Victoria L. Demetriou, Eftychia Kyriakou, Leondios G. Kostrikis

**Affiliations:** Laboratory of Biotechnology and Molecular Virology, Department of Biological Sciences, University of Cyprus, 75 Kallipoleos Avenue, P.O. Box 20537, 1678 Nicosia, Cyprus

## Abstract

Few natural intergenotypic hepatitis C virus (HCV) recombinants have been characterised, and only RF1_2k/1b has demonstrated widespread transmission. The near-full length genome sequences for two cases of 2k/1b recombinants (CYHCV037 and CYHCV093) sampled in Cyprus were obtained using strain-specific RT-PCR amplification and sequencing protocols. Sequence analysis confirmed their similarity with the original RF1_2k/1b strain from St. Petersburg, N687. These two isolates significantly contribute to the sequence data available on this recombinant and confirm its increasing spread among individuals from Eastern Europe, and its association with transmission through intravenous drug use. Phylogenetic analyses reveal clustering of the sequence 3′ to the recombination point, not seen in the topology of the 5′ sequences, implying a more complicated evolutionary history than that held to date. The increasing cases of HCV recombinant strains underline the requirement of their contribution to the standardised rules of HCV classification and nomenclature, molecular epidemiology, diagnosis, and treatment.

## 1. Introduction

The hepatitis C virus (HCV) is a single-stranded positive-sense RNA virus, belonging to the *Flaviviridae* family, and exhibits substantial global sequence diversity [1, 2]. The heterogeneity of the HCV genome has led to a proposed consensus of six genotypes (1–6) and numerous closely related subtypes (a, b, c,…) [3]. Until 2001, HCV was thought to evolve in a clonal manner, with diversity generated through the accumulation of mutations. However, several events of inter- and intragenotypic homologous recombination have been reported to date from various parts of the world [4–12]. Most of these strains appear to be isolated events and do not appear to be circulating in infected populations. A 2k/1b recombinant was found in St. Petersburg, Russia [7], thought to have emerged by homologous recombination during minus-strand synthesis via template switching [13], and seems to be circulating among intravenous drug users in Russia. Isolates of this strain, labelled RF1_2k/1b, have also been found in Ireland, Estonia, Uzbekistan, Western Siberia, and France [14–18]. 

In a recent study of HCV strains circulating in Cyprus, two putative recombinant isolates were discovered among the HCV-infected population [19], where discordance was observed between the classification of two distinct regions of the genome. Consequently, in this study, the near-full genomes of these strains have been sequenced using strain-specific protocols, and they have been confirmed as variants of RF1_2k/1b, confirming a widespread transmission of this strain, and providing more sequence data that reveal a complex evolutionary history.

## 2. Results

### 2.1. RT-PCR and Sequencing

**Figure 1:**
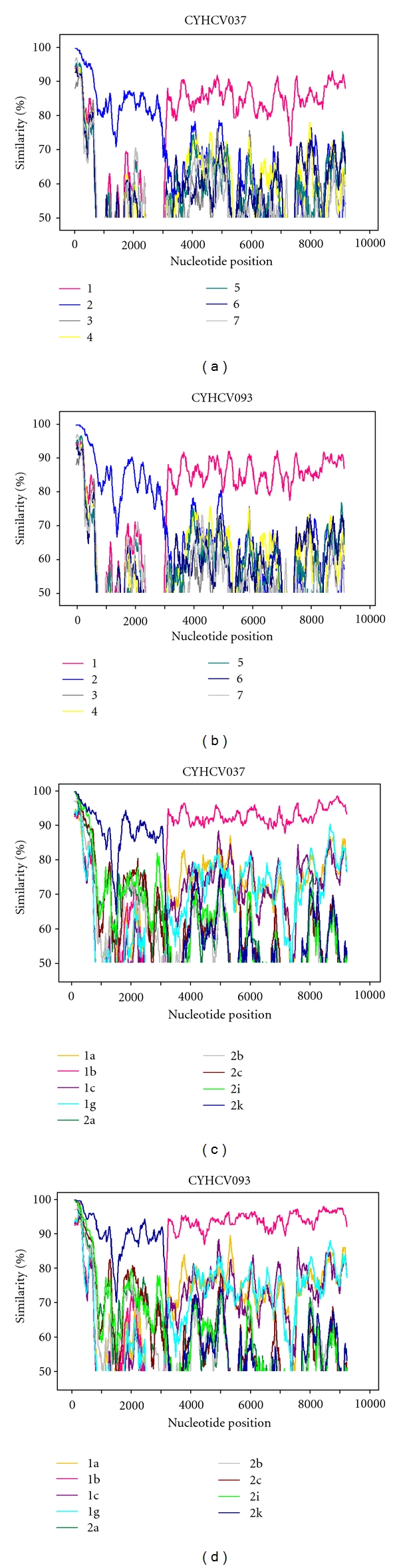
Simplot analyses of the two 2k/1b recombinant candidate strains, showing percentage sequence similarity over nucleotide position. (a, c) are for strain CYHCV037, and (b, d) for strain CYHCV093. The plots on the top are the results of a genotype-level analysis, with genotypes corresponding to line colours annotated below the plots. The bottom plots are the results of a subtype-level analysis, once the genetically closest parental genotypes had been determined using the top plots. The subtypes corresponding to the coloured lines are annotated below the plots.

**Figure 2:**
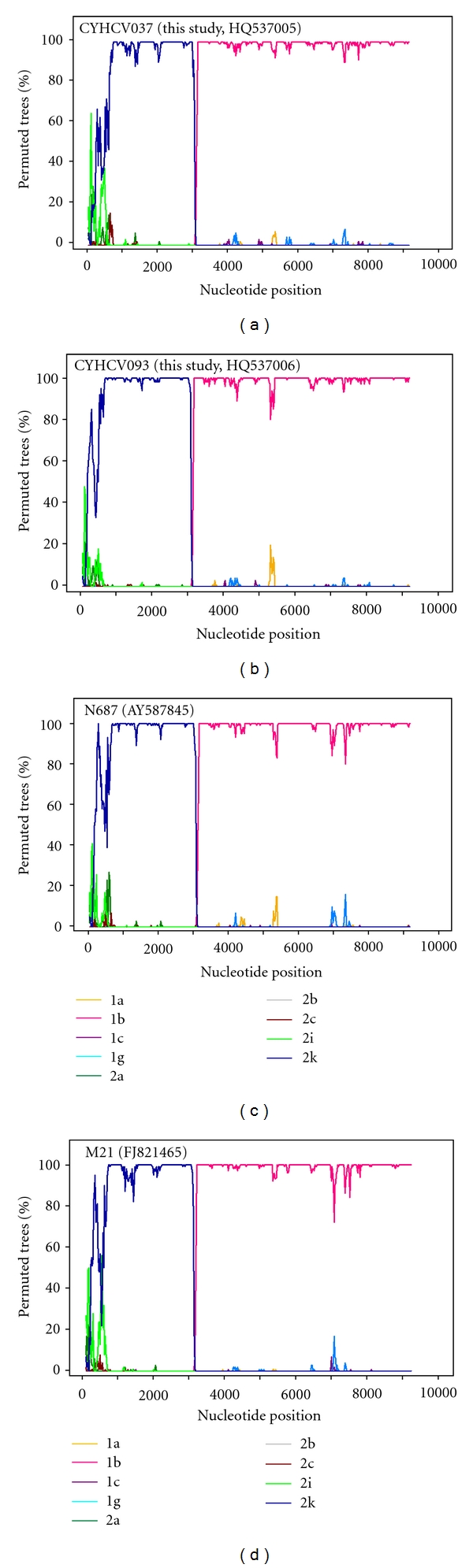
Bootscan plot of percentage permuted trees over nucleotide position across the full length of the 2k/1b recombinant strains. (a, b) are for the strains described in this study and (c, d) for the only near-full 2k/1b genomes submitted to GenBank to date. Strain identifiers are indicated at the top left corner of each plot. In brackets are the GenBank accession numbers of the sequences. The annotation below shows the HCV subtype of the consensus sequences corresponding to each line colour in the plots.

 The strain-specific near-full genome amplification and sequencing protocols were designed and successfully applied to the two strains under investigation. Due to genetic variations between the two isolates, not all PCR and sequencing primers were appropriate for both, resulting in two slightly designs (see Figure  1 for CYHCV037 and Figure  2 for CYHCV093 in Supplementary Material available online at doi: 10.1155/2011/710438). All resulting sequence readings were clear, implying no mixed infections in the samples in question. 

### 2.2. Characterisation of Recombinant Strains

 During a study of the molecular epidemiology of HCV infection in the general population of Cyprus, two samples were found to be subtype 2k in the Core-E1 region, and 1b in the NS5B region [19]. The samples were taken from two Georgian males (lab codes CYHCV037 and CYHCV093), aged 32 and 39 years at the time of sampling. The stated routes of transmission were unsafe sexual practises and intravenous drug use. 

The near-full length sequences (nt. 87-9287, positions according to H77, GenBank accession no. NC_004102) were successfully derived from the two samples from 5-6 overlapping PCR fragments. To characterise the sequences, compare them to classified reference strains, and investigate the event of recombination along the length of the sequence, SimPlot, Bootscan, and phylogenetic analyses were carried out. A Simplot graph was constructed at first on a genotype level using consensus sequences derived from references, and, following determination of the parental genotypes, on a subtype level of the parental genotypes (Figure 1). This clearly demonstrates that the strains described here are more similar to genotype 2, specifically 2k, on the 5′ side of their genomes, and more similar to genotype 1, specifically 1b, on the 3′ side of their genomes, with a point of recombination in the NS2 region, after position 3,000 (position according to strain H77). Bootscan plots were constructed for each sequence using reference sequences of the parental genotypes 1b and 2k as well as the only two available reference sequences for 2k/1b, N687 (GenBank accession number AY587845) and M21 (GenBank accession number FJ821465) (Figure 2). The results confirm that strains CYHCV037 and CYHCV093 are 2k/1b recombinant strains, with a recombination point in the same genomic location as the reference 2k/1b isolates. The nucleotide and deduced amino acid sequences of the two strains found in Cyprus were according to the original Russian N687 strain, with no insertions or deletions. Analysis by visual inspection of nucleotide and amino acid alignments in the NS2 region spanning the stated crossover point revealed a high degree of similarity between all recombinant strains reported in this study and the ones previously reported (data not shown). The two strains here are identical to the Russian N687 isolate in their partial 5′ noncoding region. Also, in the interferon-sensitivity-determining region (ISDR) of the NS5A sequence, the amino-acid sequences of the two strains in this study are identical to those of the N687 isolate, and therefore identical to the “wild-type” subtype 1b associated with interferon resistance [13, 20].

**Figure 3:**
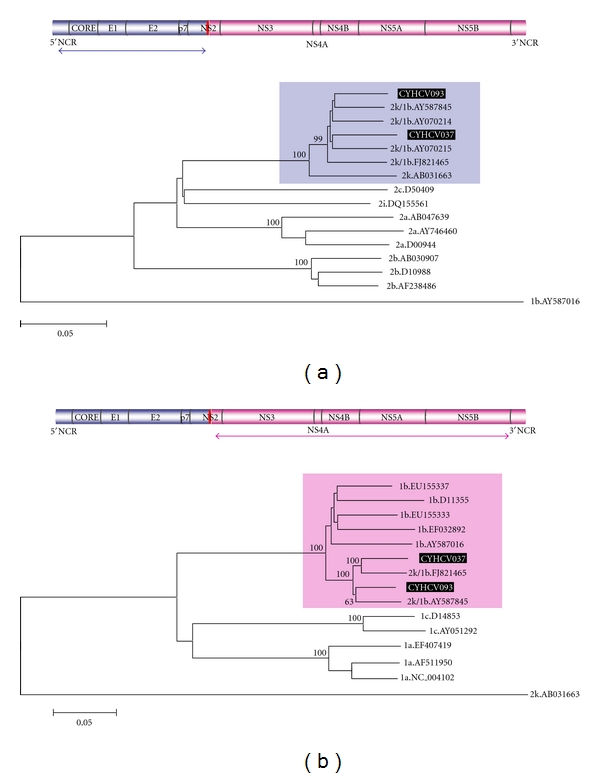
Neighbour-Joining trees for (a) the region 5′ of the recombination point and (b) the region 3′ of the recombination point, as seen by the indications on the genome maps above each tree, for the 2k/1b recombinant strains identified in this study (highlighted in black), other 2k/1b sequences obtained from GenBank, and reference stains of the corresponding parental genotypes. The trees are rooted with 1b and 2k strains, respectively. Grouping can be seen within the corresponding parental subtypes and is highlighted with coloured boxes. Numbers at branch nodes indicate the percentage bootstrap support for 1,000 replicates. The scale at the bottom left of each tree indicates the divergence between any two sequences obtained by summing the branch lengths between them.

Subsequently, phylogenetic trees were constructed for the regions on either side of the recombination point (Figure 3). Additional closely related sequences derived from HCVBLAST searches were used in the analyses and these are Acc. No. AY070214 and AY070215, both 2k/1b strains, used in the tree of the 5′ region of the genome, and EU155337 and EU155333, both 1b, used in the tree of the 3′ segment. The partial regions of the two strains described here group within the 2k and 1b subtypes, respectively, with a bootstrap value of 100. All 2k/1b recombinants cluster together in both trees, within the subtype group, with bootstrap support >98. The 1b tree (Figure 3(b)) presents grouping of CYHCV093 with strain N687 and CYHCV037 with strain M21, with bootstrap support of 63 and 100, respectively, whereas the topology of the 2k/1b cluster within the 2k subtype (Figure 3(a)) does not present any statistically significant subgroups (bootstrap support values <55).

### 2.3. Nucleotide Sequence Accession Numbers

 GenBank accession numbers for the near-full genome nucleotide sequences obtained in this study are HQ537005 for strain CYHCV037 and HQ537006 for strain CYHCV093.

## 3. Discussion

The natural 2k/1b recombinant, RF1_2k/1b, was first identified in 2002 in St. Petersburg, Russia in five intravenous drug users [7], one of which was later sequenced along the full open reading frame (isolate N687, acc. no. AY587845) [13]. Since then, several other RF1 infections have been identified in individuals from countries of Eastern Europe and former members of the Soviet Union [14–16, 18]. The most recently found isolate (M21, Acc. no. FJ821465) [17] and the original N687 strain are the only ones that have been sequenced along the complete genome to date. 

In the investigation of partial sequences from samples of the HCV-infected population of Cyprus there was evidence for two putative 2k/1b recombinant isolates. These samples were characterised in more detail by near-full genome sequencing and analysis, by designing strain-specific RT nested PCR and sequencing protocols. All resulting sequence readings were clear, implying that the strains in question are recombinants and not mixed infections of pure subtypes 2k and 1b. The results of Simplot and Bootscan analyses confirm that isolates CYHCV037 and CYHCV093 are genetically close variants of the RF1_2k/1b recombinant strain, with the crossover point in the same genomic location as the reference N687 and M21 strains. 

The epidemiological characteristics of the patients from whom the strains were derived were similar to the characteristics of the individuals carrying the RF1_2k/1b strains discovered to date. These demonstrate and confirm a trend pointing towards young men from countries of the former Soviet Union and Eastern Europe, and injecting drug use as the main risk behaviour. Hence, RF1_2k/1b appears to be circulating in small populations more than any other HCV recombinant, as several cases of infections have now been reported. The discovery of these strains in Cyprus emphasises the increasing transmission of this type in various European regions. 

As only two RF1_2k/1b genome sequences have been submitted to GenBank to date [13, 17], the sequences described here double the publicly available near-full genome sequence information for this strain. It has therefore been difficult to make conclusions regarding the molecular evolution of this strain, but with the addition of two more near-full genomes, hypotheses about this can now begin to be formulated. In the phylogenetic analyses carried out here, the recombinants group closely together forming distinct subclusters within the parental subtype group, demonstrating a low divergence between them and an evolutionary separation from the pure subtypes, in agreement with previous findings [15]. However, the RF1 also demonstrated statistically significant biphyletic separation of its isolates. This was observed for the 1b sequences on the 3′ side of the recombination point, but not for the 2k sequences on the 5′ side of the genome. It is important to note that not all 2k/1b strains found have been sequenced fully and the sequence data available for the analyses of the different regions was not the same and was limited. Only in the analysis for the sequences 5′ of the recombination site were there more strains available than those that have been sequenced along the full genome. Therefore, from the data available, the observed difference in clustering behaviour between the regions on either side of the recombination point could signify that the original recombination event took place between closely related 2k quasispecies and genetically diverse 1b species in the host, present perhaps due to multiple points of infection through unsafe injecting drug use, or due to 1b quasispecies diversity, providing a number of recombination events, and the spread from two of these is represented in the strains that have been sequenced to date. Hence, the two strains discovered in Cyprus not only provide more sequence information for the 2k/1b recombinant strain but also provide a different view on the evolutionary history of this strain than that which has been held to date. With the addition of more near-full genomes within this recombinant type, a more accurate evolutionary picture of this strain will become apparent.

The serendipitous identification of two variants of the HCV RF1_2k/1b in Cyprus was the result of a molecular investigation across the near-full genome. The results of this study highlight the opinion that the actual prevalence of this HCV type has been underestimated and that its molecular evolution is possibly more complicated than the hypothesis of one recombination point giving rise to one single common evolutionary ancestor. Epidemiologically, HCV recombinant RF1_2k/1b appears to be spreading in Europe and the Mediterranean region, through population movements of intravenous drug users. This recombinant is the most widespread and broadly distributed for HCV, highlighting the need to better investigate recombination in epidemiological studies, to include it in the considerations for the criteria of HCV classification and nomenclature, and to give greater consideration to their diagnosis and clinical management. 

## 4. Methods

### 4.1. Samples

 During the study of the molecular epidemiology of HCV infection in the general population of Cyprus, discrepancies were found in the subtype classification of two genomic regions for two samples, which were found to be subtype 2k in the Core-E1 region, and 1b in the NS5B region [19]. These strains were considered putative 2k/1b recombinants and were therefore selected for near-full genome sequencing. The samples were taken from two consenting Georgian male patients. Patient CYHCV037, 32 years old when sampled in 2005, had been first diagnosed HCV-seropositive in 2003 and stated sexual transmission as the possible route of infection. Patient CYHCV093, 39 years old when sampled in 2007, had been diagnosed HCV-seropositive in 2000 and stated intravenous drug use as the transmission route. Neither individual had taken antiviral therapy at the time of sampling.

### 4.2. Experimental Design

 Protocols (Supplementary Figures 1 and 2) and primers (Supplementary Table  1) for RT nested PCR and sequencing of overlapping fragments was designed based on a published protocol [11]. PCR primers were designed to be subtype- or strain-specific, by using alignments of available 2k/1b, 2k, and 1b sequences from the Los Alamos HCV sequence database (http://hcv.lanl.gov/content/index). Inner PCR primers were used for initial sequencing of PCR products and further strain-specific sequencing primers were subsequently designed by “primer walking”, using the resulting sequences. With the assumption that the strains would have the same recombination point as the known 2k/1b recombinant, where the crossover point was found to be around nucleotide 3164 (numbered according to strain H77) [13], primers on the 5′ side of this point were designed to be 2k-specific and primers on the 3′ side were designed to be 1b-specific. Also, the region covering this point was sequenced bidirectionally from three different PCR products to ensure the agreement of sequences.

### 4.3. RNA Extraction, RT-PCR, and Near-Full Genome Sequencing

Viral RNA was extracted from 200 *μ*L plasma previously extracted at the initial sampling of the patients [19], using the High Pure Viral RNA kit (Roche Diagnostics, Manheim, Germany), following the manufacturer's instructions. 

Initially, one-step RT-PCR reactions were set up using the Superscript III one-step RT-PCR system with Platinum Taq high fidelity (Invitrogen, Carlsbad, CA, USA) and the outer forward and reverse primers as previously described [19]. For the nested PCR reactions, 1X Platinum PCR SuperMix High Fidelity (Invitrogen, Carlsbad, CA, USA) was used with 3 *μ*L of the corresponding RT-PCR product, and 20 pmol each of the inner forward and reverse PCR primers. PCR products were visualised after electrophoresis on a 2% agarose gel and purified using the QIAquick PCR Purification Kit (Qiagen, Hilden, Germany). 

Cycle sequencing reactions were performed on 1 *μ*L of each secondary PCR products using the BigDye Terminator system v3.1 (Applied Biosystems, Warrington, UK) with 5 pmol of each sequencing primer (Supplementary Table  1), as described previously [19]. Direct population sequencing was carried out in order to obtain consensus sequences reflecting the dominant quasispecies population in each sample. Sequences were read using the ABI 3130 Genetic Analyzer and obtained through the ABI sequencing analysis 5.2 software (Applied Biosystems, Foster City, CA, USA). The results were manually looked over and polymorphisms where double peaks were observed at equal intensity in the chromatogram were annotated according to standard IUB base codes. All resulting sequences of each strain were used for stitching together the near-full genome (nt 87-9287, numbered according to strain H77).

### 4.4. Characterisation of Recombinant Strains

 Simplot and Bootscan analyses were carried out on the resulting sequences to visualise the percentage sequence similarity of each isolate to reference sequences along the length of their sequence and investigate recombination. The near-full genome sequences were aligned with reference strains representing all HCV genotypes and all available subtypes of the appropriate corresponding genotypes in each case in MEGA v4 [21] and uploaded into SimPlot v3.5.1 software (http://sray.med.som.jhmi.edu/SCRoftware/). The analysis also included the two available 2k/1b full genome sequences, N687 (GenBank Acc. no. AY587845) and M21 (GenBank Acc. no. FJ821465). The analyses were carried out with windows of 200 bases proceeding in steps of 20 bases, using the Kimura 2-parameter distance model, and a Neighbour-Joining tree model with 100 bootstrap replicates.

Phylogenetic analyses were carried out separately on the genomic regions on either side of the crossover point (positions 87-3150 and 3200-9287, resp., numbered according to H77), in order to confirm the classification of each region in the corresponding subtypes, by including reference strains and the most closely related (>90% similarity) sequences derived from an HCVBLAST search (http://hcv.lanl.gov/content/sequence/BASIC_BLAST/basic_blast.html). Sequences were aligned in MEGA v4 [21], where a Neighbour-Joining tree was constructed as described previously [19].

For visual inspection and sequence comparison, the nucleotide sequences of the strains described here were uploaded into MEGA v4, aligned with isolates N687 and M21, and automatically converted to their translated protein sequences.

### 4.5. Reference Sequences

 The GenBank accession numbers of the reference strains used in the construction of phylogenetic trees described above are seen in the corresponding figures in the results section. For the Simplot and Bootscan analyses, the GenBank accession numbers of the reference sequences used are as follows: 1a.NC_004102, EF407419, AF511950; 1b.AY587016, D11355, EF032892; 1c.D14853, AY051292; 2a.AB047639, AY746460, D00944; 2b.AB030907, AF238486, D10988; 2c.D50409; 2i.DQ155561; 2k.AB031663; 2k/1b.AY587845; 2k/1b.FJ821465; 3a.X76918, AF046866, D17763; 3b.D49374; 3k.D63821; 4a.NC_009825, DQ418788; 4d.DQ418786, DQ516083; 5a.AF064490, NC_009826; 6a.DQ480513, Y12083; 6b.D84262; 6c.EF424629; 7a.EF108306.

## Supplementary Material

Schematic diagrams of the experimental designs for amplification and sequencing of recombinant HCV 2k/1b strains.
