# Supplementary figures and images for: Near-Full Genome Characterisation of Two Natural Intergenotypic 2k/1b Recombinant Hepatitis C Virus Isolates
Source: Adv Virol. 2011 May 15;2011:710438. doi: 10.1155/2011/710438 (PMC3270303; doi:10.1155/2011/710438)

## 2k/1b – PCR and sequencing design for CYHCV037

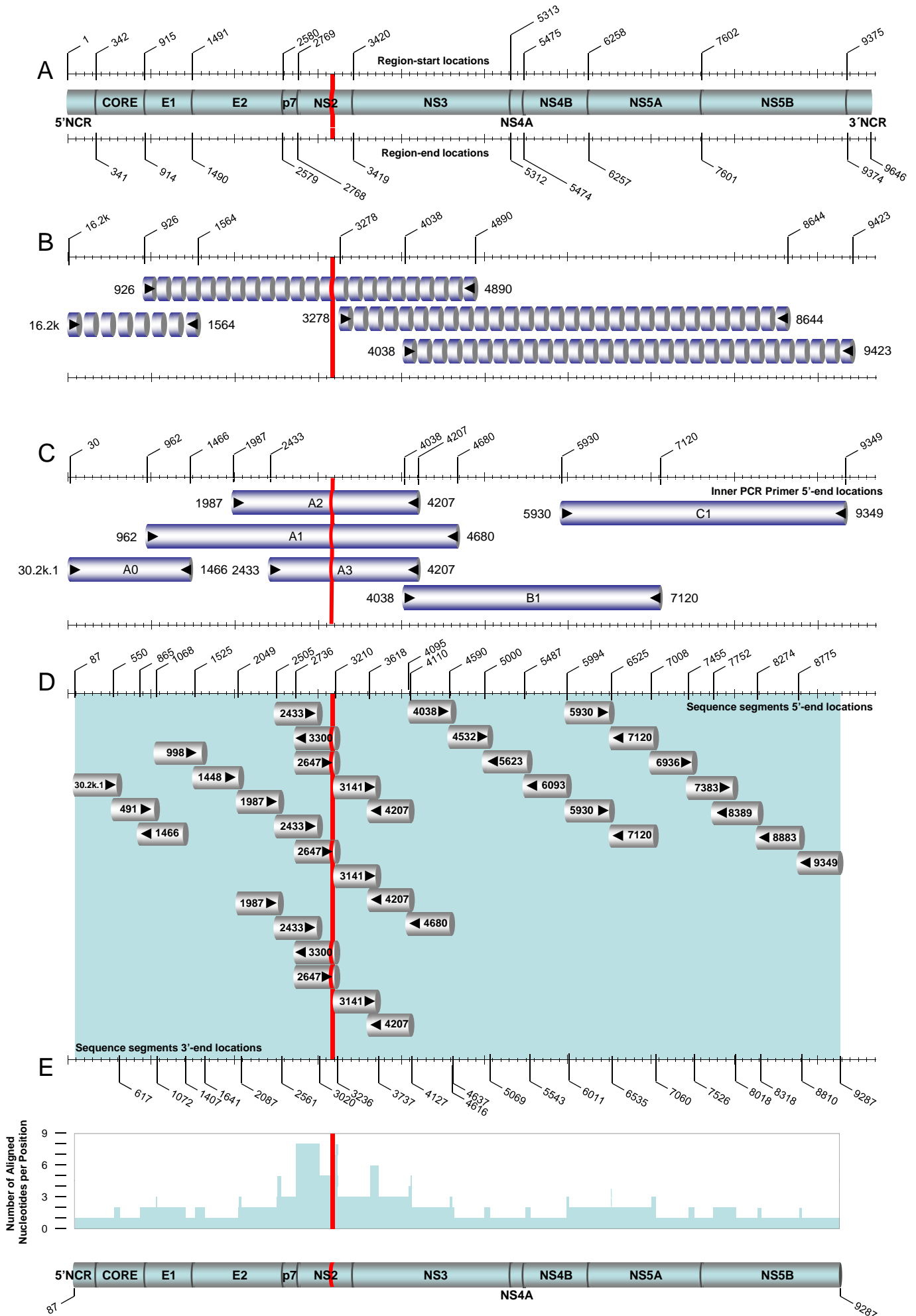

Supplementary figure 1

Supplement: Supplementary file 1 — Schematic diagrams of the experimental designs for amplification and sequencing of recombinant HCV 2k/1b strains. [file 710438.f1.pdf]

## 2k/1b – PCR and sequencing design for CYHCV093

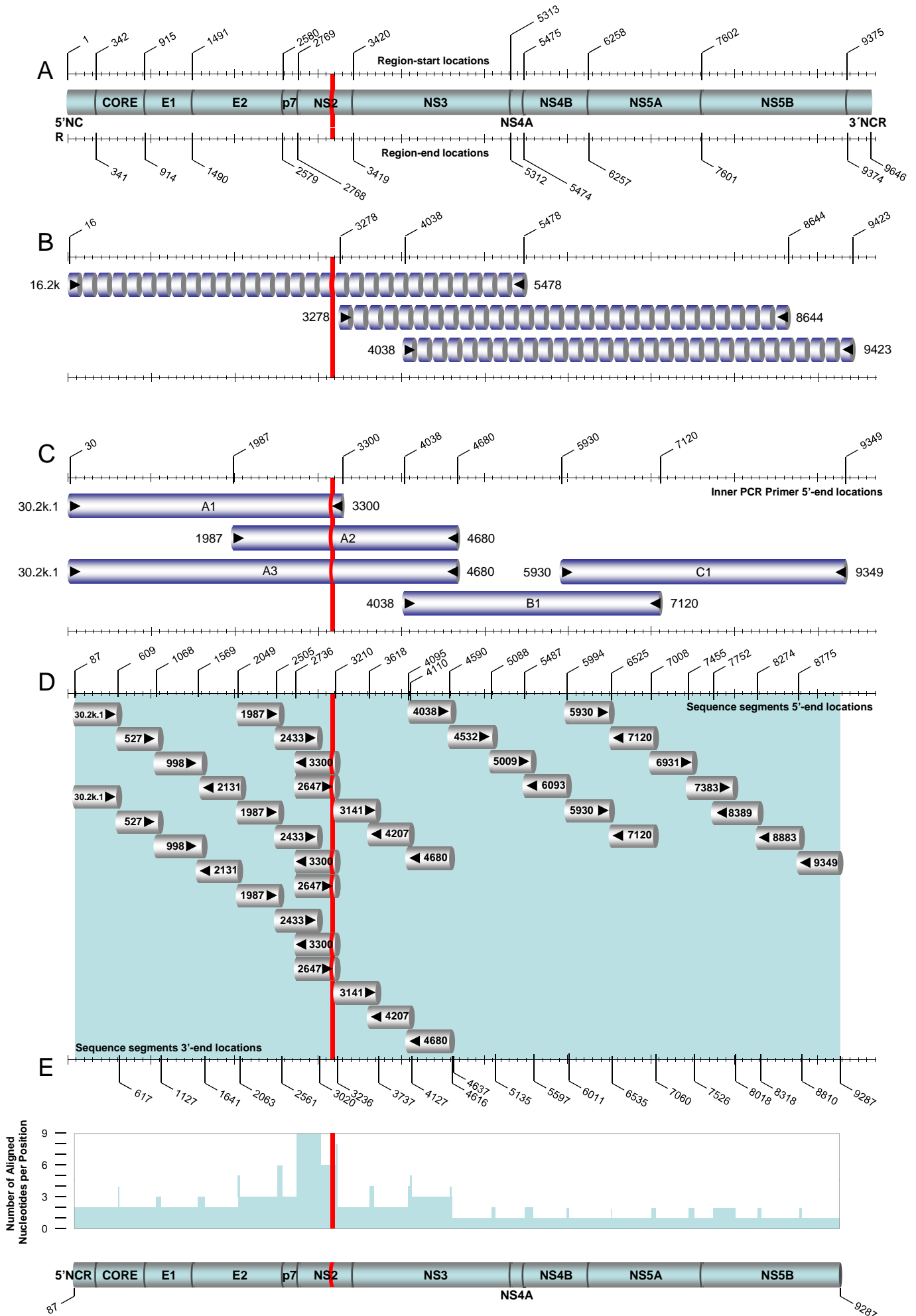

Supplementary figure 2

Supplement: Supplementary file 2 [file 710438.f2.pdf]
