# Supplementary material for: Near-Full Genome Characterisation of Two Natural Intergenotypic 2k/1b Recombinant Hepatitis C Virus Isolates
Source: Adv Virol. 2011 May 15;2011:710438. doi: 10.1155/2011/710438 (PMC3270303; doi:10.1155/2011/710438)
Supplement: Supplementary file 3 [file 710438.f3.pdf]

Supplementary table 1. PCR and sequencing primers

Designation [a]	*Sequence* [b]	*Position* [c]
**PCR primers**		
16.2k (F) [d]	GGGGCGACACTCCGC	16-30
30.2k.1 (F) [d,e]	CCATGAATCACTCCCCTGTGAGG	30-52
926 (F)	GAACACCAGTCAGACCTACATGG	926-948
962 (F)	CTCTAACAGCAGCATCACGTGG	962-983
1466 (R) [e]	GAGGATGACGACGACCTTGG	1447-1466
1564 (R)	GAAAAGGCTGGTAAGGCGAAGC	1543-1564
1987 (F) [d,e]	GGTTTGGATGCACATGGATGAATGG	1987-2011
2433 (F) [e]	GACGTGCAGTACTTGTATGGCC	2433-2454
3278 (F) [d]	GGAGACCAAGATCATCACCTGGG	3278-3300
3300 (R) [d,e]	CCCAGGTGATGATCTTGGTCTCC	3278-3300
4038 (F) [d,e]	GGCAGCGGCAAGAGCAC	4038-4054
4207 (R) [e]	GCACCTGTGGTGATGGTCC	4189-4207
4680 (R) [d,e]	CGCCGGTRWAGCCCGTC	4664-4680
4890 (R)	GGACCGAGGAGTCAAACATGC	4870-4890
5478 (R) [d]	AGGCGCACTCTTCCATYTCATC	5457-5478
5930 (F) [d,e]	CGTGGCCTTTAAGGTYATGAGCG	5930-5952
7120 (R) [d,e]	CGRAGCGGGTCRAAAGAGTC	7101-7120
8644 (R) [d]	GCTGAGTACCTAGTCATAGCCTCCG	8620-8644
9349 (R) [d,e]	ACCCCTACAGAAAGTAGGAGTAGGC	9325-9349
9423 (R) [d]	AAAAAACAGGATGGCCTATTGGCC	9397-9423
**Sequencing Primers**		
491 (F)	GAAGACTTCCGAGCGGTCC	491-509
527 (F)	CCAGCCCATCCCTAAAGATCG	527-547
998 (F)	GGTACTACATGTCCCTGGATGC	998-1019
1448 (R)	CAAGGTCGTCGTCATCCTCC	1448-1467
2131 (R)	CATTTGGTGTAAGTAGCCTCAGGG	2108-2131
2647 (F)	CCATCTTCTTCATAGCTGCCTGG	2647-2669
3141 (F)	GTCCAGATGATGCTGCTAACCC	3141-3162
4532 (F)	GAAGAAATGTGATGAGCTCGCTGC	4532-4555
5009 (F)	GTTCTGGGAGAGCGTCTTCAC	5009-5029
5623 (R)	CAGAAGGTCTCAAGGGCTCG	5604-5623
6093 (R)	GGTTCATCCACTGCACAGCC	6074-6093
6931 (F)	CCCTGGCTAGCTCTTCTGC	6931-6949
6936 (F)	GCCAGCTCTTCAGCTAGTCAG	6936-6956
7383 (F)	GCTACAAAGACCTTCGGCAGC	7383-7403
8389 (R)	CCGATATAAAGTCGCTCTGTGAGC	8366-8389
8883 (R)	GAGTCATCAGAATCATCCTTGCCC	8860-8883

[a] Orientation of the PCR primer is indicated in parenthesis: F, forward; R, reverse.

[b] IUB base codes annotate equimolar mixtures of bases in the oligonucleotides: Y, C and T; R, A and G; W, A and T.

[c] Positions are numbered according to HCV strain H77, GenBank accession no. NC_004102.

[d] Modified from Noppornpanth *et al.*, 2006; all other primers were designed *de novo* for this study.

[e] Also used as sequencing primers.
